# Toward a Personalized Real-Time Diagnosis in Neonatal Seizure Detection

**DOI:** 10.1109/JTEHM.2017.2737992

**Published:** 2017-09-11

**Authors:** Andriy Temko, Achintya Kr. Sarkar, Geraldine B. Boylan, Sean Mathieson, William P. Marnane, Gordon Lightbody

**Affiliations:** Department of Electrical and Electronic Engineering and Irish Centre for Fetal and Neonatal Translational ResearchUniversity College CorkT12 P2FYCorkIreland; Department of Electronic SystemsAalborg University9220AalborgDenmark; Department of Paediatrics and Child Health and INFANT CenterUniversity College CorkT12 P2FYCorkIreland; Academic Research Department of NeonatologyInstitute for Women’s Health, University College LondonLondonWC1E 6AUU.K.

**Keywords:** Neonatal, seizure, detection, online adaptation

## Abstract

The problem of creating a personalized seizure detection algorithm for newborns is tackled in this paper. A probabilistic framework for semi-supervised adaptation of a generic patient-independent neonatal seizure detector is proposed. A system that is based on a combination of patient-adaptive (generative) and patient-independent (discriminative) classifiers is designed and evaluated on a large database of unedited continuous multichannel neonatal EEG recordings of over 800 h in duration. It is shown that an improvement in the detection of neonatal seizures over the course of long EEG recordings is achievable with on-the-fly incorporation of patient-specific EEG characteristics. In the clinical setting, the employment of the developed system will maintain a seizure detection rate at 70% while halving the number of false detections per hour, from 0.4 to 0.2 FD/h. This is the first study to propose the use of online adaptation without clinical labels, to build a personalized diagnostic system for the detection of neonatal seizures.

## Graphical abstract

Personalized detector of neonatal seizures through on-the-fly unsupervised adaptation of a generic model improves detection accuracy.

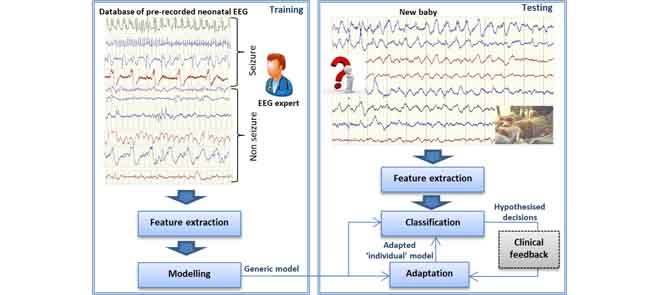

## I. Introduction

Individual healthcare decisions [1] empowered by technological solutions such as automatic diagnostic systems have been shown to be more accurate than more generic systems in many areas of biomedical signal processing. These systems are built using the data of a targeted user/patient which eliminates the inter-subject variability of the training data and allows the system to focus on learning intra-subject characteristics, thus simplifying the estimation and recognition problem. Well-known examples include subject-specific brain computer interfaces [2], patient-specific epilepsy detection systems [3] and patient-specific diagnostic consultation However, in the development of an EEG-based seizure detector for the newborn [5], the EEG data of the baby cannot be obtained before the baby is born. The successful system must be able generalize over the pre-recorded and pre-annotated data from other babies to be able to detect seizures from the data of the new baby and alarm the clinical personnel. There are also significant clinical pressures for the availability of useful information from EEG monitoring, within hours of birth.

A number of research groups [5]–[13] have previously developed neonatal seizure detection algorithms (SDA) in an attempt to assist healthcare professionals with objective decision support. A typical SDA comprises of the following main stages: i) The signal representation stage (feature-level) – where relevant features are robustly extracted from the pre-processed EEG signal. ii) The classification stage (classifier level) – where the extracted feature or feature vectors are assigned to the seizure or non-seizure class using a set of rules and thresholds which are either automatically derived from the data (classifier) [5], [8], [13] or manually adjusted following or mimicking the reasoning of expert neurologists [6], [7], [9], [12]. iii) The post-processing stage (decision level) – this involves both temporal smoothing to reduce noise and possibly other transformations that may offer some support in the decision making process to a clinician.

A notable improvement has recently been achieved in neonatal seizure detection with the development of a Support Vector Machine (SVM) based neonatal seizure detector [14], [15] which has completed a pre-market European multi-centre clinical investigation^1^ to support its regulatory approval and clinical adoption [16]. This system utilises an SVM classifier trained on a high dimensional set of extracted features that carry temporal, frequency, structural and energy information about the neonatal EEG. The analysis of algorithmic performance in [17] and [18] revealed that the performance of the detector is significantly correlated with seizure duration, amplitude, rhythmicity and the number of EEG channels involved in the seizure during peak seizure activity.^1^https://clinicaltrials.gov/ct2/show/NCT02160171, https://clinicaltrials.gov/ct2/show/NCT02431780

The combination of various (even many) classifiers has been widely researched both theoretically in the literature [19] and practically through public competitions such as Kaggle [20]. The underlying principle here is that performance improvement may be obtained from the diversity of classification methods; popular classifier combinations include blending, bagging, boosting, stacking, etc. Such classifier ensembles tend to yield good results when there is a significant diversity among the models. This diversity can come from inherent algorithmic randomness (like random decision trees) or from a deliberate difference in the optimisation functions used in training (such as the difference between the discriminative SVM and the generative GMM) or the usage of different training datasets. For example, consider a situation in which there are two seizure classifiers; to make this conceptually easy, consider that one classifier utilises the EEG and the other uses video. If the EEG based classifier just misses a seizure, whilst the other classifier confidently identifies a seizure based on some video cues, it would seem sensible that given its confidence, the video based classifier becomes the expert at this particular moment and can therefore guide the EEG based classifier to better performance over this data. Each classifier is looking at the problem in a different way and may contribute complementary expertise.

**FIGURE 1.**
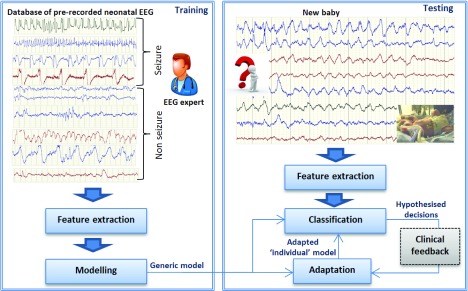
From generic to personalized neonatal seizure detector. The adaptation of the generic model can be performed on the fly. The optional clinical feedback in the testing stage can be used to purify the hypothesised decisions.

In neonatal intensive care units (NICU), neonates that are suspected of developing neurological complications can be continuously monitored using EEG for several days; indeed, pre-term infants can often be monitored over a period of a few weeks. Seizure characteristics can vary between neonates and thus long EEG recordings can be exploited, as shown in Fig. 1, to derive individualized models from generic patient-independent systems. Such a subject adaptive system must capture the specifics of the monitored neonate on-the-fly in order to improve its performance; however, data annotations are required to drive this optimisation. One way to achieve this is to utilize clinical feedback for the small number of suspected events (alarms) and re-build or adapt the model to each specific newborn, incorporating the new annotated information. EEG experts are generally not available during unsociable hours and NICU staff typically lack EEG training and many feel unsupported in the interpretation of neonatal EEG [21]. Since the purpose of building the automated SDA is to provide continuous objective brain monitoring that will typically produce alerts when the clinical expertise is not available – this solution may not be practical. An alternative solution is to allow the detector to learn from its own decisions, balancing the gain obtained from new patient-specific information with the uncertainty of its automated hypothesised labels.

A patient-adaptive neonatal SDA is proposed here which combines generic and personalized seizure detectors. Previous work has demonstrated that different classifiers (even when trained on the same data) can provide useful complementary performance to each other for the neonatal patient independent seizure detection task. The novelty of this current study is that a patient adaptive classifier is proposed that will adapt and improve its performance to a specific neonate over the first hours after birth, without the need for clinical labels. A pre-trained patient independent SVM-based system that was developed in [5] and [14] is used to automatically provide labels for data from a new unseen baby which can then be utilised to adapt a Gaussian Mixture Model (GMM) [22] based detector to achieve improved personalised performance, in real time. To the best of our knowledge, this work provides the first application of adaptive personalised neonatal seizure detection without the need for clinical input – this provides state of the art performance for neonatal seizure detection.

## II. Materials and Methods

### A. Dataset

The database is composed of EEG recordings from 18 full-term newborns recruited in the Neonatal Intensive Care Unit (NICU) of Cork University Maternity Hospital (CUMH), Cork, Ireland. The CareFusion NicOne video EEG monitor was used to record multi-channel EEG at 256Hz using the modified 10–20 system of electrode placement with the following 8 EEG bipolar channels F4-C4, C4-O2, F3-C3, C3-O1, T4-C4, C4-Cz, Cz-C3 and C3-T3. All electrographic seizures were annotated independently by an experienced neonatal neurophysiologist (GB) using simultaneous video EEG. The combined length of the EEG recordings totalled 816.7 hours with per patient mean/median length of 45.4/48.5 hours and contained 1389 electrographic seizures. The dataset contains a wide variety of seizure types, including both electrographic-only and electro-clinical seizures of focal, multi-focal and generalized types. The continuous EEG recordings were not manually edited to remove the large variety of artifacts and poorly conditioned signals that are commonly encountered in long EEG recordings in the real-world NICU environment. An additional dataset of 55 non-seizure babies (1 hour per baby) is used to augment the representation of the EEG background activity. This small dataset is used only for training. The described dataset is used to evaluate the developed algorithms retrospectively.

For performance evaluation, the leave-one-out (LOO) procedure was followed where all but one patients’ data are used for training and the remaining patient’s data are used for testing. The procedure is repeated until each of the 18 patients has been a test subject and the mean results are reported. LOO is known to be an unbiased estimation of the true generalization error [23]. Additionally, the LOO eliminates any subjectivity from the test protocol, hence it can be repeated and exactly the same results will be obtained.

This dataset is truly representative of the real-life situation in the NICU and it allows for a robust estimate of the algorithm performance. In fact, the LOO performance estimated on this dataset was shown to closely match the performance which was independently assessed on a separate large clinical dataset, as reported in [14] and [18].

### B. Patient Independent Seizure Detection Algorithms (PI-GMM, PI-SVM, PI-FUSION)

The typical patient-independent SDA consists of the following blocks: EEG signal pre-processing, feature extraction, modelling, post-processing and decision making. Three patient independent classification algorithms have been developed employing: the support vector machine classifier (PI-SVM), a Gaussian mixture model based classifier (PI-GMM) and a fusion of the two classifiers (PI-Fusion). More details on these SDAs including the list of extracted features and the probabilistic interpretation of the classifier output can be found in Appendices A-D. These SDAs as described in [14] and [22] are patient independent systems, which have no prior sight of the EEG of the neonate under test. Importantly, these systems have been developed so that every channel of the EEG is processed separately and independent of the other channels, which means that the system is robust to the number and choice of channels.

Both the GMM and SVM classifiers perform an extraction of a compact representation of the training data for each class. For the GMM this is based on the data centroids which are obtained by averaging over the training data. In the case of the SVM however, this is based on a subset of the training data which lies close to the discriminative boundary. While the support vectors are selected from the training data in the context of both classes, the GMM centroids are however class-indifferent, and thus are not optimized to increase the separability of the problem. The previous work on combining SVM and GMM classifiers for neonatal seizure detection showed a significant disparity between classifier decisions, resulting in an agreement of only approximately 50% of the false positives [22].

A simple *blending* of patient-independent GMM and SVM classifiers using the geometric mean is used to provide the patient independent SDA, (PI-FUSION):

\begin{equation*} P_{PI-Fusion}=\sqrt {P_{PI-GMM}P_{PI-SVM}} \end{equation*}

Here $P_{PI-GMM}$ and $P_{PI-SVM}$ are the probabilities of seizure provided by the patient independent GMM and SVM classifiers respectively. The geometric mean combination was found more suitable than the arithmetic mean for fusing classifiers with different probability density functions [20]. In fact, the SVM probabilities usually follow a gamma distribution and the GMM posteriors follow a Gaussian distribution.

### C. Oracle Systems – Patient Dependent SDAs Using Patient Specific Clinical Labels (PD-SVM and PD-GMM)

The patient dependent seizure detectors, PD-SVM and PD-GMM, were constructed in the same way as their patient-independent alternatives, but with a small portion (a few minutes) of the test patient data (with ‘true’ clinical neurophysiologist annotations) used in training. These systems, PD-SVM and PD-GMM are referred to as Oracle systems, as they use labels provided by a clinical expert. These systems are used to estimate the theoretical performance improvements that could be obtained if some clinical labels were available for the test patient. In our case, the Oracle systems were not fully patient-dependent as they were still trained with data from other patients (not just with the targeted patient’s data). Moreover, no special emphasis was given to the sampling of the targeted patient data – the new data were simply randomly mixed with the existing training data. These resultant systems were then tested on the remaining *unseen* data from that specific test patient – the systems are therefore no longer patient-independent as they have seen samples of seizure and non-seizure activities from the targeted testing patient.

Patient-dependent SDAs are quite popular in the adult population (especially those that are based on intracranial EEG), where the data collected during previous hospital visits are annotated and used to develop patient-specific models for subsequent visits [24], [20]. In fact, it has been shown in [13] that a *fully* patient-dependent neonatal SDA performs much better than a patient-independent one. However, in the neonatal population such systems are impossible as EEG data from the newborn brain is not typically recorded until the baby is born. There is a significant clinical time pressure – the detection system should be functioning and supporting clinical decisions from the moment the EEG electrodes are placed on the newborn’s scalp, literally within a few hours of birth.

The Oracle systems used here mimic a scenario where small portion of annotated data of the testing subject is available beforehand: For example when a clinician (a neurophysiologist) who is alerted by an alarm generated by the generic neonatal SDA (SVM or GMM), is able to provide feedback about the true label of this alarm (as shown in Fig. 1). The label (seizure or non-seizure) can then be used to adapt automatically the models with this new information, thus making the models personalised.

### D. A Semi-Supervised Learning Scheme for Patient Adaptive SDA (PA-GMM and PA-FUSION)

**FIGURE 2.**
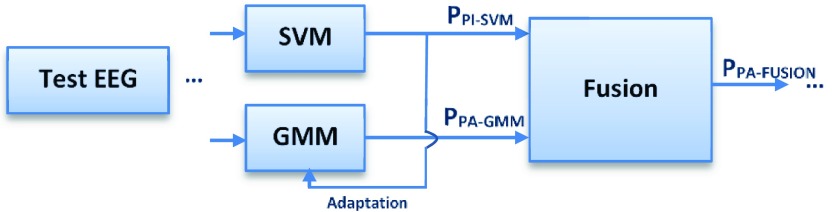
The ensemble of patient-adaptive GMM and patient-independent SVM SDAs.

This section details an alternative technique for the *blending* of the two classifiers where the PI-SVM system is used to automatically label new data, for example from a new unseen patient – these labels can then be used for the on-line adaptation of a GMM based detector (PA-GMM). The final ensemble (PA-FUSION) consists of the fixed patient-independent PI-SVM classifier and the changing patient-adaptive PA-GMM classifier, which are then blended, as shown in Fig. 2.

The SVM paradigm has achieved considerable success in a wide variety of problems in a batch setting where all of the training data is available in advance. Several learning techniques have been developed to facilitate SVM training over very large datasets, however, only a few have been proposed for incremental, online and active learning. Most of active learning techniques, such as adiabatic learning [25], are approximate and require several passes through the data to reach convergence. Although these algorithms allow for training on large datasets that is significantly faster than typical state-of-the-art SVM solvers, they are still incapable of real-time training and allow for little (or no) control over the confidence of the new data during the training procedure. This results in the limited use of these methods in an online setting suitable for real-time medical applications [26].

In contrast to the SVM, several well-established techniques exist to perform online learning for a Gaussian mixture model based classifier. These are widely used in the area of speech processing, for instance to improve the speech recognition accuracy by adapting the generic phonetic models to those of a specific speaker [27], [28].

#### 1) MAP Adaptation of the GMM Model

Given an ordered set of $N$ new feature vectors $\mathbf {X}=\left \{{ \boldsymbol {x}_{1},\ldots , \boldsymbol {x}_{N} }\right \}$, a corresponding ordered set of the associated seizure probabilities produced from the PI-SVM, are generated as, $P_{S-SVM}=\left \{{P_{S-SVM,1},P_{S-SVM,2},\ldots ,P_{S-SVM,N} }\right \}$. The seizure and non-seizure GMMs, are parameterised by $\theta _{S}=\left \{{ \mu _{S,j},w_{S,j},\Sigma _{S,j}: \forall j\in \left \{{ 1,2,\ldots ,M_{S} }\right \} }\right \}$ and $\theta _{NS}=\left \{{ \mu _{NS,j},w_{NS,j},\Sigma _{NS,j}: \forall j\in \left \{{ 1,2,\ldots ,M_{NS} }\right \} }\right \}$, respectively, (see Appendix C). The patient-adaptive models are then developed by the adaptation of the original patient-independent models based on new test patient data. The conventional maximum a-posteriori (MAP) adaptation is used here and consists of the following steps:

a) Compute the occupational likelihood for each feature vector, ${x}_{i}$, with respect to the $m^{\mathrm {th}}$ Gaussian component of each class model $\theta _{C}$. The occupational likelihood determines how relevant a particular new feature vector is to the given Gaussian component,
  \begin{equation*} P_{C,m}\left ({ x_{i},\theta _{C} }\right )=\frac {w_{C,m}g\left ({ x_{i}\vert \mu _{C,m},\Sigma _{C,m} }\right )}{\sum _{j=1}^{M_{C}} {w_{C,j}g\left ({ x_{i}\vert \mu _{C,j},\Sigma _{C,j} }\right )} }. \end{equation*}
b) Compute the mean of the adaptation data, weighted by the occupational likelihood, over the $N$ new feature vectors, **X**. For the $m^{\mathrm {th}}$ Gaussian component of class $C$; this yields,
  \begin{equation*} E_{C,m}\left ({ \mathrm {\mathbf {X}} }\right )=\frac {\sum _{i=1}^{N} {P_{C,m}\left ({ \boldsymbol {x}_{i},\theta _{C} }\right )\boldsymbol {x}_{i}} }{\sum _{i=1}^{N} {P_{C,m}\left ({ \boldsymbol {x}_{i},\theta _{C} }\right )} }. \end{equation*}
c) Update the new mean of the $m^{\mathrm {th}}$ Gaussian component of class $C$ as the weighted average of the original mean and the adaptation data mean,
  \begin{equation*} \mu _{C,m}\leftarrow \alpha \mu _{C,m}+\left ({ 1-\alpha }\right )E_{C,m}\left ({ \mathrm {\mathbf {X}} }\right ). \end{equation*}

In this manner every single mean component of the models for each class are updated based on the weighted average of the original mean and the mean of the adaptation data weighted by the occupational likelihood. Three iterations are used in the MAP adaptation routine in this work.

#### 2) Novel MAP Algorithm Based on the Confidence of the Automated Labels

When applying MAP adaptation, a label is required for the new feature vector to indicate which class specific model should be adapted with these new data. These labels are generated automatically using the PI-SVM based SDA. Such labels could be generated by thresholding the SVM probabilities, for example with a threshold of 0.5. However, the choice of any specific threshold would have a significant effect on the performance of the system and as such would therefore require careful tuning. Moreover, in this approach, the data would be split between the two class specific models – that is, a given chunk of data would be used to adapt either the seizure model or the non-seizure model, depending on its assigned label. Additionally, as seizures are relatively rare events, it is likely that the seizure model would not be adapted at all during the first few hours for a new patient. In order to maximise the power of the data available from a new patient, the MAP adaptation was modified to enable the use of all the new testing data to adapt both the seizure and non-seizure GMM models, simultaneously.

First, the data set of $N$ new feature vectors are grouped into clusters according to their associated PI-SVM probabilistic output. The grouping is performed by partitioning the probability space [0 1] into a set of non-overlapping bins; the $k^{\mathrm {th}}$ bin will have a lower limit $\underline {P_{k}}$ and an upper limit $\bar {P_{k}}$. Given the ordered set of SVM probabilities $P_{S-SVM}=\left \{{ P_{SVM,1},P_{SVM,2},\ldots ,P_{SVM,N} }\right \}$, the corresponding ordered set of $N$ new feature vectors, $\mathrm {\mathbf {X}}=\left \{{ x_{1},x_{2},\ldots ,x_{N} }\right \}$ is then clustered for the seizure model using the rule, $i\in I_{S,k}~\mathrm {if}~\underline {P_{k}}\le P_{SVM,i}<\bar {P_{k}}$, where $I_{S,k}$ is the indicial set for the $k^{\mathrm {th}}$ cluster, for seizure model adaptation. The $N$ new feature vectors would also be clustered for the non-seizure model using the complementary rule, $i\in I_{NS,k}~\mathrm {if}~\underline {P_{k}}\le \left ({ 1-P_{SVM,i} }\right )<\bar {P_{k}}$, where $I_{NS,k}$ is the indicial set for the $k^{\mathrm {th}}$ cluster, for the non-seizure model adaptation. These clusters represent different confidences of being relevant to a chosen class.

The MAP adaptation algorithm can now be reformulated as a weighted combination of the statistics of each of the K groups, with the weight-set, $\left \{{ \beta _{1},\beta _{2},\ldots ,\beta _{K}}\right \}$,

\begin{equation*} \mu _{C,m}\leftarrow \alpha \mu _{C,m}+\sum _{k=1}^{K} \beta _{k} \left ({ \frac {\sum _{i\in I_{C,k}} {P_{C,m}\left ({ \boldsymbol {x}_{i},\theta _{C} }\right )\boldsymbol {x}_{i}} }{\sum _{i\in I_{C,k}} {P_{C,m}\left ({ \boldsymbol {x}_{i},\theta _{C} }\right )} } }\right ), \end{equation*}

where

\begin{equation*} C\in \left \{{ S, NS }\right \}. \end{equation*}

The weights sum up to 1; the weight of the original data, $\alpha =1-\sum \nolimits _{k=1}^{K} \beta _{k} $, determines how aggressive the adaptation will be on the new data.

**FIGURE 3.**
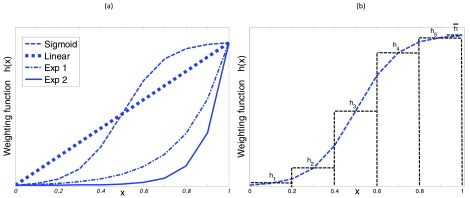
(a) Four different weight functions; here x = PSVM for providing weights for updates to seizure class GMM, x = (1-PSVM) for updating non-seizure class GMM. (b) An example of a sampled sigmoid weighting using 5 clusters.

Intuitively, the cluster with a higher $\bar {P_{k}}$ should have a larger gain, $\beta _{k}$. The four basic and intuitive weighting schedules shown in Fig. 3 (a) are investigated. The group weights, $\left \{{ \beta _{1},\beta _{2},\ldots \beta _{K} }\right \}$, for both classes follow either of a simple straight line, a sigmoid, or two exponential functions each with a different decay rate. In all cases the weights increase monotonically with confidence. In contrast to the linear function which gives linearly decaying weights to the groups as confidence reduces, the sigmoid function nonlinearly emphasises the high-confidence groups and attenuates the low confidence groups. The two exponential functions are more conservative than the linear and sigmoid functions, in that they allow for the adaptation of the class-specific models to be focussed only on new data where the label confidence is high.

The number of confidence-based data groups is first chosen and the continuous probability space [0, 1] in Fig. 3 (a) is then partitioned to provide $K$ clusters. Fig. 3 (b) shows how for example a sigmoid is sampled to provide the weights for 5 clusters. Every point in the $k^{\mathrm {th}}$ cluster is assigned the same central weight $h_{k}$; after sampling these weights are then normalized to provide a partition of unity,

\begin{equation*} \beta _{k}=\frac {h_{k}}{\bar {h}+\sum _{k=1}^{K} h_{k} }. \end{equation*}

The weight of the original data, $\alpha $, is always the largest and effectively places more confidence on the original model for which the training data (with clinical labels) are more certain.

The resultant patient-adaptive GMM system (PA-GMM) is then blended with the patient-independent SVM classifier (PI-SVM) using the Eq. 1 to form the final ensemble (PA-FUSION).

### E. Performance Evaluation

The system performance is measured as the average area under the receiver operating characteristic curve (AUC) [29]. The AUC is calculated by plotting the sensitivity vs specificity values computed over the probabilistic output produced for every epoch of EEG. The area under the ROC curve is an effective way of comparing the performance of different systems – a random discrimination will give an area of 0.5 under the curve while perfect discrimination between classes will give unity area under the ROC curve. Additionally, the AUC90 is reported where the area under the curve is computed for a specificity larger than 90%. The AUC90 is more reflective of the potential clinical scenario as it focuses and quantifies the performance in the area with very low false detection rates.

**FIGURE 4.**
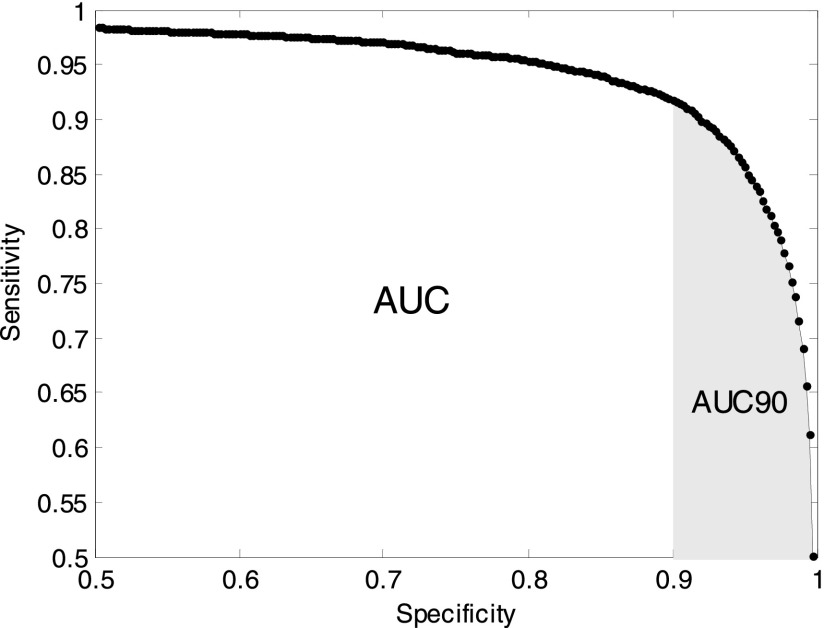
Performance of a SDA – measured as the area under the ROC curve.

Fig. 4 shows an example of the ROC curve for a typical SDA from which AUC and AUC90 can be computed. The mean AUC area across all patients is reported in this study. The ROC area is related to the Wilcoxon test of significance [30]. This relationship can be used to derive statistical properties of the ROC area such as its standard error and to calculate the statistical significance in the performance of two algorithms (ROC areas) evaluated on the same data; this takes into account the correlation of the two ROC curves [14], [30], [36]. The details of the statistical test can be found in Appendix E.

## III. Experimental Results

Table 1 summarises the performance scores of the various systems: Baseline patient independent systems (PI-SVM, PI-GMM and PI-FUSION), the Oracle systems using some clinical labels for each test baby (PD-SVM, PD-GMM) and the Adaptive systems in which automated labels are generated, (PA-GMM and PA-FUSION).

**TABLE 1:** Performance of the Neonatal Seizure Detection Systems

		AUC (%)	AUC90 (%)
Baseline	PI-GMM	95.70	78.40
	PI-SVM	96.50	82.60
	PI-FUSION	96.62	82.61
Oracle	PD-GMM	97.51	86.33
	PD-SVM	97.17	85.80
Adaptive	PA-GMM	96.91	82.60
	PA-FUSION	97.03	84.10

The SVM patient-independent system (PI-SVM) on this dataset yields a performance of 96.50% and 82.6% for AUC and AUC90, respectively. The GMM patient-independent system (PI-GMM) on this dataset yields a performance of 95.70% and 78.49% for AUC and AUC90, respectively. The performance of a simple blending of GMM and SVM (PI-Fusion) results in a performance of 96.62% and 82.65% for AUC and AUC90. As can be seen, the performance of the ensemble (PI-FUSION) is slightly improved in comparison with the best single patient-independent classifier (PI-SVM), which has an AUC of 96.50%.

The performance of the Oracle patient-dependent system, PD-SVM, is 97.17% and 85.80%, for AUC and AUC90, respectively. The performance of the Oracle patient-dependent system, PD-GMM is 97.51% and 86.33% for AUC and AUC90, respectively.

The adaptive GMM system (PA-GMM) provides a performance which is an improvement over its patient-independent GMM counterpart (PI-GMM) – 96.91% vs 95.70% for AUC, and 82.6% vs 78.4% for AUC90. In fact, it also outperforms the best baseline patient-independent SVM system (PI-SVM), 96.91% vs 96.50% for AUC, with the same performance as measured by AUC90.

**FIGURE 5.**
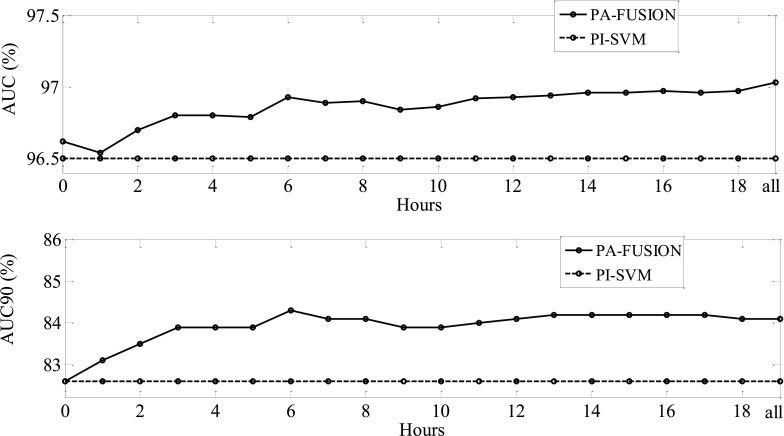
Performance of the baseline SVM and adaptive fusion system measured as AUC (top) and AUC90 (bottom) as a function of time.

Fig. 5 shows how the mean AUC and AUC90 (determined over all the unseen records in the LOO performance assessment routine) evolve with training time, as compared to the baseline PI-SVM performance. In this experiment, the data from each unseen baby within the LOO validation scheme is split into one hour segments. The PA-GMM is then adapted sequentially on each hour of data, until eventually all the data was used. The labels were produced automatically using the PI-SVM – an exponential weighting function (exp2) was used with 10 groups. The performance of the fused classifier PA-FUSION was reported after each hour of adaptation – this was evaluated over the whole recording from the beginning, to allow for a fair comparison with the baseline performance. This procedure was repeated for each baby within the LOO scheme, where for each unseen test baby the GMM was re-initialised to the PI-GMM. The last point on the curves in Fig. 5 indicates the offline performance of the fused classifier, that is, it provides the performance of a retrospective corrected viewing of the unseen EEG recording. Similarly, the first point on the curves in Fig. 5 represents the performance of the PI-FUSION system which is simply the geometric mean combination of the baseline PI-SVM and PI-GMM, when adaptation has not yet been performed. This evaluation strategy mimics the real-life operation of the neonatal SDA – the system runs in real-time but it may re-adjust or correct its previous decisions based on the evidence observed.

Table 2 shows the effect the choice of the weights on the adaptive systems. Here four different weight profiles were considered: linear, sigmoidal, an exponential decay, and a more aggressive exponential decay curve. Table 3 presents the performance of the proposed patient adaptive systems for different numbers of data clusters.

**TABLE 2:** Performance With Different Adaptation Weighting Functions

		Function	AUC (%)	AUC90 (%)
Adaptive	PA-GMM	Sigmoid	96.70	81.6
		Linear	96.79	82.3
		Exp 1	96.85	82.9
		Exp 2	96.91	82.6
	PA-FUSION	Sigmoid	96.97	83.8
		Linear	96.99	84.0
		Exp 1	97.02	84.2
		Exp 2	97.03	84.1

**TABLE 3:** Performance With Different Number of Groups

		# Groups	AUC (%)	AUC90 (%)
Adaptive	PA-GMM	8	96.93	82.5
		10	96.91	82.6
		15	96.90	82.7
	PA-FUSION	8	97.03	84.0
		10	97.03	84.0
		15	97.03	84.1

**FIGURE 6.**
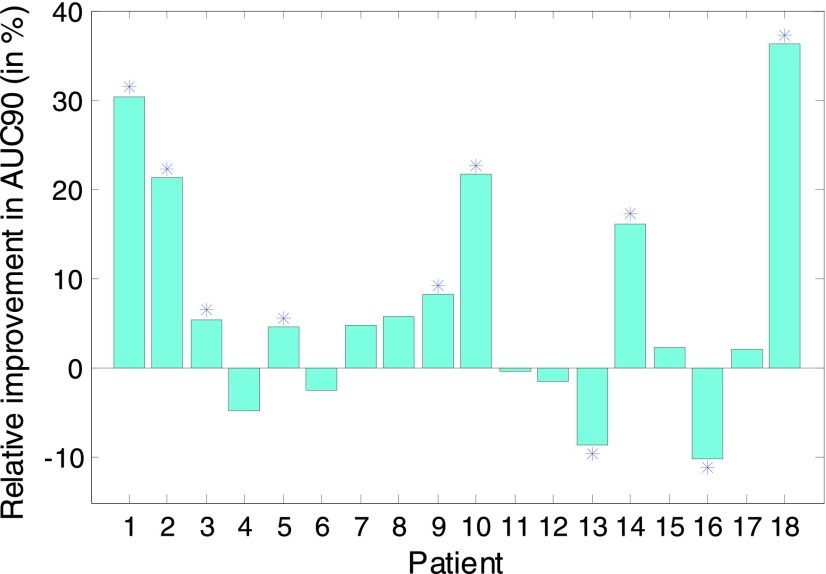
Relative improvement in AUC90 between the PA-FUSION and PI-FUSION systems. ‘^*^’ indicates statistical significance at á set to 1%.

Fig. 6 shows the relative improvement in AUC90 for each subject in the dataset between PA-FUSION and PI-FUSION, to illustrate the effect of adaptation. The relative improvement is calculated as (AUC90PA-FUSION – AUC90PI-FUSION)/ (1 – AUC90PI-FUSION). The statistically significant difference at $\alpha $ set to 1% as in [14] is indicated with the asterisk.

## IV. Discussion

### A. Performance of the Oracle Systems (PD-SVM and PD-GMM)

This work explores the unsupervised use of patient specific test data to improve the developed models. It can be seen that both PD-SVM and PD-GMM yield performance improvements as compared with their patient-independent counterparts. The SVM system improved its AUC from 96.50% to 97.17% and its AUC90 from 82.6% to 85.80% The GMM system improved its AUC from 95.70% to 97.51% and its AUC90 from 78.49% to 86.33%. Comparing the improvements and the absolute performances of the Oracle systems from Table 1, it can be seen that PD-GMM is more sensitive to the new data and better exploits even small amounts of the targeted patient data. The GMM based classifier is also much easier to adapt because of the availability of the well-established model adaptation routines – this is highlighted by the performance improvement achieved for the GMM. As a comparison, however, the baseline SVM patient-independent SDA achieved a better performance (96.50% vs 95.70% for AUC, and 82.6% vs 78.49% for AUC90) when compared with the patient independent GMM – this shows that for our experiments that the SVM provided better performance than the GMM when dealing with batch data; this is also confirmed in [22].

An important conclusion that can be extracted from the performance of the Oracle systems is the upper bound on the performance of automatic adaptive systems. In fact, what the automatic adaptive SDA does is to create a system-generated label to avoid consulting a clinician (or to keep functioning in the absence of a neurophysiologist) and to use this label to adapt the models. As no SDA is error-free, the use of the true label can be seen as an estimate of the maximum achievable performance given the chosen methodology.

### B. Performance of the Patient-Adaptive Systems (PA-GMM and PA-FUSION)

The results of the proposed adaptive SDAs demonstrate the ability of the proposed method to capture the test patient specifics by the automatic, unsupervised (in the sense that there is no human involved), on-the-fly adaptation of the GMM models using well-established adaptation routines as a core. The combination of PI-SVM and PA-GMM as an adaptive fusion system (PA-FUSION) provided comparable and improved results in AUC and AUC90 values in comparison with the GMM adaptive system alone (PA-GMM), 97.03% vs 96.91% for AUC, and 84.1% vs 82.6% for AUC90. This indicates that the PA-GMM system still carries complementary information to the PI-SVM system which is exploited with the geometric mean fusion – even though it was adapted based on the automatic labels provided by the PI-SVM classifier.

The Oracle systems achieve the best possible results. The comparison of the adaptive systems with the Oracle indicates that the adaptive SDA performance is close to that of the human-supervised adaptation (Oracle). We can also conclude that the use of a relatively small amount of manually-annotated data can lead to better performance than unsupervised adaptation with a lot of data – this is not unexpected given that the decisions produced by the PI-SVM classifier contain errors; these errors inevitably affect the model purity.

### C. How the Performance of Patient Adaptive Systems (PA-GMM and PA-FUSION) Depend on the Duration of Adaptation to a Specific Patient

From Fig. 5 it can be appreciated that the performance of the adaptive system increases rapidly during the first 6 hours of recording with little or no additional performance improvement after this time. Since the adaptation is driven by the PI-SVM system, the PA-GMM adaptive system gradually changes its nonlinear decision boundaries to approximate those of the SVM system – thus improving the GMM performance for data for which the SVM was confident. However, it is essential that the GMM is adapted to effectively fill in the gaps in its performance, rather than to be over-trained to fully mimic the PI-SVM. Therefore, as shown in Fig. 5, the similarity between the outputs of the two classifiers increases with increasing data which then leads to a slow-down in the progress of the adaptive fusion system (PA-FUSION). The adaptation data allow the GMM adaptive system to learn the specifics of the test patient at the cost of incorporating into its models the errors that come with the imperfect SVM hypotheses. At the same time, the amount of the adaptation data for the GMM adaptive system can be seen as a trade-off between learning new information (and thus being more accurate under the above-mentioned constraints) and being different (and thus complementary) to the SVM baseline. It can be seen that the in-built intrinsic difference between the two classifiers such as the generative GMM and the discriminative SVM allows the adaptive fusion system to benefit from adaptation and to maintain a stable performance even until the end of the recording.

Our previous work has demonstrated that the performance of the PI-SVM and PI-GMM systems had a standard deviation of AUC of ~2% across all iterations of the LOO validation [22]. The performance of the presented patient-adaptive method does not therefore depend on the group of patients used in the training of the PI-SVM but rather on the specifics of the testing patient – the amount of seizure data present within the first few hours of recording.

### D. The Influence of the Weight Function and the Number of Data Groups on the Performance of PA-GMM And PA-FUSION Systems

The weights in Eq. 5 and Eq. 6 control the trade-off between the gain of learning new patient-specific information and the cost of introducing noise into the models – non-seizure characteristics to the seizure model and seizure characteristics to the non-seizure model. It can be seen that the GMM adaptive systems (PA-GMM) with any of these weighting profiles provided better performance than the PI-GMM system (results in Table 1, AUC = 95.70%). The aggressive exponential decay weighting (Exp2) provides the best trade-off (AUC = 97.03% as compared to 95.7%). This is a conservative weighting function – the faster the decay, the lower the influence of low-confidence groups in the adaptation framework. For the number of clusters, the performance was observed to be stable in the tested range (5 – 25 clusters).

It is worth noting that the parameters of the system were not tuned to reach the best possible performance. The aim of this study was to demonstrate the potential of the unsupervised on-line adaptation for improved performance in neonatal SDA through the personalisation of detection algorithms. Further performance improvements should be possible, if instead of sampling common generic weighting functions, the weights could instead be estimated on the training data using maximum likelihood optimisation, for example.

### E. Translational Relevance and Statistical Significance

From Fig. 6 the comparison between patient-adaptive and patient-independent ensemble systems can be performed with the test of statistical significance (Appendix E). Both systems represent a fusion of PI-SVM and either PI-GMM or PA-GMM. The significance level to reject the null hypothesis was set to 1% ($\alpha =0.01$). It can be seen that the adaptation improves the AUC90 significantly in 8 patients and decreases the performance in 2 patients. In 4 out of 8 patients with the improved AUC90, this increase was above 20%. In the remaining 8 patients, the performance, as measured in AUC90 with the chosen cut-off point, does not change significantly. The average relative improvement in AUC90 in those patients whose AUC90 changed significantly is approximately 10%.

Event based metrics provide a better measure of the clinical benefits of the adaptive fusion system. After 7 hours of unsupervised adaptation for each unseen patient in the LOO scheme, the average seizure detection rate (over all unseen babies) improved from 63% to 70%, while keeping a fixed false detection rate of 0.2 FD/h (on average 1 false detection every 5 hours). This corresponds to an additional 90 detected seizures detected over the whole database. Alternatively, focusing on the reduction of the FD/h rate, while maintaining a seizure detection rate at 70%, the number of false detections per hour was halved, from 0.4 FD/h to 0.2 FD/h.

**FIGURE 7.**
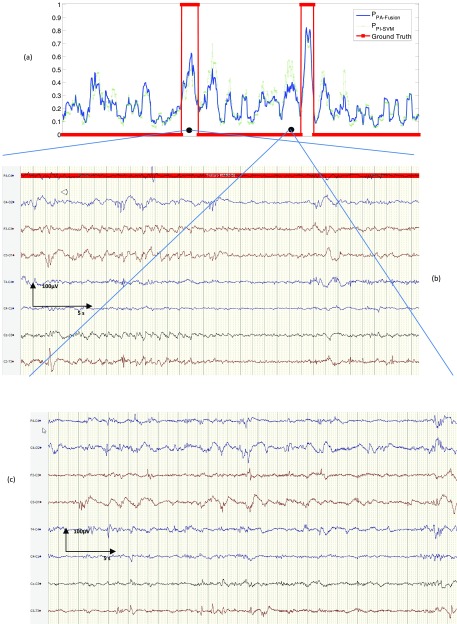
Real-time functioning of the developed personalised SDA algorithm. A) Probabilistic output of patient-adaptive and patient-independent SDAs with superimposed ground truth. B) 30s of seizure activity detected with higher probability with adaptive fusion system. C) 30s of non-seizure activity detected with lower probability.

An example of the real-time functioning of the developed algorithm is shown in Fig. 7, where the probabilistic output of the PA-FUSION SDA is contrasted with the probabilistic output of the baseline PI-SVM SDA for 1 hour of EEG with the superimposed clinical annotations. It can be seen that both seizures in the example result in a higher probability output from the PA-Fusion system, whereas the probabilistic output for the non-seizure EEG in-between is attenuated. This improvement comes from learning patient-specific information in an unsupervised way which makes the system more robust to inter-patient variability and allows it to focus the learning process on the difference between seizure and non-seizure characteristics.

It is important to realise that from an event detection and hence clinical point of view, this technique provides performance improvements. If a decision threshold of 0.5 was utilised in Fig. 7, then both systems would detect the two seizure events shown; however there would be 4 false alarms with the PI-SVM approach which would be reduced to 1 false alarm with the proposed patient adaptive approach. If the threshold was increased to 0.6, the PI-SVM approach would miss one seizure event with the PA-FUSION approach still detecting the 2 events. There would now only be one false detection for the PI-SVM detector; this was improved to zero false detections using the proposed PA-Fusion detector.

## V. Conclusions

This study has contributed to the implementation of personalised healthcare in the area of seizure detection in the newborn. A combination of patient adaptive generative and patient independent discriminative classifiers has led to an improvement in the detection of neonatal seizures over the course of long EEG recordings, as validated on a long unedited EEG dataset. More accurate detection comes from both the different nature of the classification approaches and the real-time incorporation of patient-specific data. To the best of our knowledge, this is the first study to propose the use of online adaptation to build a personalized diagnostic system for detection of neonatal seizures.

## Figures and Tables

**FIGURE 8.**
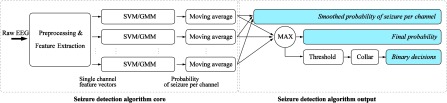
Neonatal seizure detection system diagram.

## Appendix A EEG Pre-Processing and Feature Extraction

The EEG is filtered with a band-pass zero-phase filter [0.5–13Hz] and down-sampled from 256 to 32 Hz. A 55-dimensional feature vector is extracted from an 8-second long single-channel EEG epoch, with 50% overlap. The feature set considered have been shown to be useful for neonatal seizure detection in a number of papers from different groups [14], [31] and can capture temporal, frequency, structural and energy information about neonatal seizures. The features extracted are listed in Table 4. More details can be found in [14]. The feature vectors are normalized by subtracting the mean and dividing by the standard deviation to assure commensurability of various features. These 110 normalisation constants (55 means and 55 standard deviations) are estimated on the training data and applied to the test data.

**TABLE 4:** Extracted Features of Neonatal EEG

Domains	Feature List
Frequency	• Total power (0–12Hz) • Peak frequency of spectrum • Spectral edge frequency (80%, 90%, 95%) • Power in 2Hz width subbands (0–2Hz, 1–3Hz, …10–12Hz) • Normalised power in subbands • Wavelet energy
Time	• Non-linear line length • Number of maxima and minima • Root mean squared amplitude • Hjorth parameters • Zero crossings (raw epoch, $\Delta $ and $\Delta \Delta )$ • Autoregressive modeling error (model order 1–9) • Skewness • Kurtosis • Nonlinear energy • Variance ($\Delta $ and $\Delta \Delta$)
Information theory	• Shannon entropy • Singular value decomposition entropy • Fisher information • Spectral entropy

## Appendix B An SVM Based Patient Independent Classifier

The SVM-based patient independent neonatal SDA is shown in Fig. 8 and is described in detail in [5] and [14]. For a test vector $\boldsymbol {x}\in R^{d}$, and $y\in \left \{{ -1,+1 }\right \}$, the output decision of a support vector machine is given by:

\begin{equation*} y\left ({ \boldsymbol {x} }\right )=\mathrm {sgn}\left ({ f\left ({ x }\right ) }\right ) \end{equation*}

where

\begin{equation*} f\left ({ x }\right )=b+\sum \nolimits _{j\in Q_{SVM}} {\alpha _{j}y_{j}K\left ({ \boldsymbol {x},\tilde {\boldsymbol {x}}_{j} }\right )} \end{equation*}

Here $Q_{SVM}$ is the indicial set of the retained vectors from the input training data which are called support vectors. The $j^{\mathrm {th}}$ support vector, $\tilde {\boldsymbol {x}}_{j}$, has an associated training label, $y_{j}$, and a non-zero weight $\alpha _{j}$; $b$ determines the offset of the separating hyperplane from the origin.

The Gaussian kernel is used:

\begin{equation*} K\left ({ \boldsymbol {x},\tilde {\boldsymbol {x}}_{j} }\right )=exp\left ({ -\frac {1}{2\sigma ^{2}}\left ({ \boldsymbol {x}-\tilde {\boldsymbol {x}}_{j} }\right )^{T}\left ({ \boldsymbol {x}-\tilde {\boldsymbol {x}}_{j} }\right ) }\right ) \end{equation*}

The SVM model was trained on data with per-channel seizure and non-seizure annotations, thus resulting in a single generic SVM classifier that can be applied to any EEG channel from any patient. This classifier was trained to maximise the margin, with softening of this margin included to allow a balance between decision errors and over-fitting. In the dual form, the training algorithm can be stated as the following optimisation problem over the training set consisting of $N_{T}$ input feature vectors, $\left \{{ \boldsymbol {x}_{1},\boldsymbol {x}_{2},\ldots ,\boldsymbol {x}_{N_{T}} }\right \}$ with associated labels $\left \{{ y_{1},y_{2},\ldots ,y_{N_{T}} }\right \}$ where $y_{i}\in \left \{{ -1,+1 }\right \}$.

\begin{align}&\hspace {-0.6pc}\max \limits _{\alpha }\left ({ \sum _{i=1}^{N_{T}} {\alpha _{i}-\frac {1}{2}\sum _{i=1}^{N_{T}} \sum _{j=1}^{N_{T}} {\alpha _{i}\alpha _{j}y_{i}y_{j}K\left ({ \boldsymbol {x},\tilde {\boldsymbol {x}}_{j} }\right )} } }\right )\notag \\&s.t.~\sum \nolimits _{i=1}^{N_{T}} {\alpha _{i}y_{i}=0 \quad \text {and}~0\le \alpha _{i} \le C,\quad i=1,2,\ldots ,N_{T}}\notag \\ {}\end{align}

The regularisation constant $C$ helps to control the degree of over-fitting.

Cross-validation on the training data is used to select suitable model parameters for the regularisation constant C and the Gaussian kernel width $\sigma $. During the testing stage, the features extracted from each EEG channel of multi-channel EEG are fed into the trained model so that the SVM output is generated separately for each EEG channel. The output of each SVM is converted to posterior probabilities as explained in Appendix D and then smoothed using a $15^{\mathrm {th}}$ order moving average filter which corresponds to a span of ~1 minute of EEG. Depending on the user interface chosen, the output of the system can be either per-channel probabilities, or a single probability trace (by taking the maximum of probabilities across channels), or binary decisions which are produced by applying a threshold to the final probability followed by a collar to compensate for the delay introduced by the moving average filter.

## Appendix C A Generative Approach – Using the Gaussian Mixture Model

The GMM is a generative approach in which the class-specific probability density functions over the $d$ dimensional feature space are first modelled using a weighted sum of $M_{C}$ Gaussian components, for each class $C$,

\begin{align} p_{c}\left ({ \boldsymbol {x}\vert \theta _{C} }\right )=&\sum _{j=1}^{M_{C}} \frac {w_{C,j}}{\sqrt {\left ({ 2\pi }\right )^{d}\left |{ \Sigma _{C,j} }\right |} }\notag \\&\times \,exp\left ({ -\frac {1}{2}\left ({ \boldsymbol {x}-\mu _{C,j} }\right )^{T}\Sigma _{C,j}^{-1}\left ({ \boldsymbol {x}-\mu _{C,j} }\right ) }\right )\notag \\=&w_{C,j}g\left ({ \boldsymbol {x}\vert \mu _{C,j},\Sigma _{C,j} }\right ) \end{align}

In the neonatal seizure detection problem presented here it is assumed that there are only two classes, $C\in \left \{{ non-seizure~\left ({ NS }\right ),~seizure~(S) }\right \}$. Again $\boldsymbol {x}\in R^{d}$ is a feature vector, the mixture weights for class C are $w_{C,j}$, $j=1,2,\ldots ,M_{C}$. The $j^{\mathrm {th}}$ Gaussian component for class $C$ is a $d$ dimensional Gaussian function with mean vector $\mu _{C,j}$ and covariance matrix $\Sigma _{C,j}$. The set of $M_{c}$ mean vectors weights and covariance matrices then form the parameter set $\theta _{C}$ for class $C$.

In contrast to [22], the training procedure of the seizure and non-seizure models is different in this study. First, a so-called Universal Background Model (GMM-UBM) [32] is constructed by training on all the available training data: seizure, non-seizure, artefactual. This step does not require any annotations and can benefit from the vast amount of data that is available which do not have annotations. The concept of the GMM-UBM has evolved from the speaker identification area where it was used to create a general model of human speech [32] from which a more accurate model of a targeted speaker could be derived. Similarly, in this case, the seizure and non-seizure models are derived from the GMM-UBM using Maximum a Posteriori (MAP) adaptation based on labelled class-specific data [33]. The main advantage of building seizure and non-seizure models through the GMM-UBM is that the class models inherit from the GMM-UBM the wide diversity of signal characteristics across all possible EEG states that would not have been modelled with the direct training of individual models. In effect, this controls the response of the models to ‘other’ EEG activity. If this activity is equally distanced from both seizure and non-seizure models its effect will be the same and hence it will not contribute to the decision making.

Principle Component Analysis (PCA) is first applied on the features to de-correlate them and reduce the dimensionality. This allows a diagonal covariance matrix to be used in the GMM. In the PCA transformation, 99% of the cumulative energy of the original space is retained whilst reducing the original 55 dimensional feature space to typically 20–25 dimensions (this depends on the training set used in the leave-one-out scheme). The 64 Gaussian UBM model was trained using the Expectation Maximisation (EM) algorithm, [33], [34]. Class specific models were then trained, one for seizure and one for non-seizure.

In the testing stage, the feature vectors of the test EEG data are scored against the seizure and non-seizure models and the output likelihood values are then converted into probabilities using Bayes’ theorem. If equal priors are assumed, then similar to Eq. 7, the decision boundary for the GMM can be expressed as,

\begin{equation*} y\left ({ \boldsymbol {x} }\right )=\mathrm {sgn}\left ({ f\left ({ x }\right ) }\right ) \end{equation*}

where

\begin{equation*} f\left ({ x }\right )=\ln {P_{S}\left ({ \boldsymbol {x}\vert \theta _{S} }\right )}-\ln {P_{NS}\left ({ \boldsymbol {x}\vert \theta _{NS} }\right )} \end{equation*}

The same post-processing stage, as is utilised for the SVM system is used with the GMM classifier as shown in Fig. 8.

## Appendix D A Probabilistic Output for SVM and GMM Classifiers

To determine the class membership of each input vector both classification algorithms calculate a weighted Gaussian distance (a similarity measure) from a test feature vector to each class, represented by either the Gaussian centroids for GMM or the support vectors for SVM. The output of the GMM and SVM can be converted to a probability of seizure using a sigmoid function, in the form:

\begin{equation*} P\left ({ S\vert f\left ({ \boldsymbol {x} }\right ) }\right )=\frac {1}{1+exp\left ({ \lambda f\left ({ \boldsymbol {x} }\right )+\varepsilon }\right )} \end{equation*}

where $f(\boldsymbol {x})$ is provided for the SVM and the GMM classifiers using Eq. 7 and Eq. 11, respectively. For the GMM classifier, Bayes theorem provides $\lambda = - 1$ and $\varepsilon = 0$. For the SVM classifier, the parameters $\lambda $ and $\varepsilon $ are trained using gradient descent over a subset of the training data [35].

## Appendix E A Comparison of Aucs for Two Algorithms

The ROC area is related to the Wilcoxon test of significance [14], [30], [36]. This relationship can be used to derive statistical properties of the ROC area such as its standard error (SE):

\begin{align}&SE( \gamma )\notag \\&=\sqrt {\frac {\gamma ( 1\!-\!\gamma )\!+\!( n_{A}\!-\!1 )( Q_{1}\!-\!\gamma ^{2} )\!+\!(n_{N}\!-\!1)(Q_{2}\!-\!\gamma ^{2})}{n_{A}n_{N}}} \end{align}

where $\gamma $ is the ROC area, $Q_{1}=\gamma /(2-\gamma )$ and $Q_{2}=2\gamma ^{2}/(1+\gamma )$, $n_{A}$ and $n_{N}$ are the numbers of seizure (abnormal) and non-seizure (normal) epochs. To calculate the statistical significance of a difference of the performance (AUCs) of the two algorithms evaluated on the same data, we compute the z statistic by taking into account the correlation of the two ROC curves [36]:

\begin{equation*} z=\frac {\gamma _{1}-\gamma _{2}}{\sqrt {{SE(\gamma _{1})}^{2}+{SE(\gamma _{2})}^{2}-2rSE(\gamma _{1})SE(\gamma _{2})} } \end{equation*}

where $\gamma _{1}$ and $\gamma _{2}$ refer to the observed areas associated with algorithm 1 and 2, respectively. Here, $r$ represents the estimated correlation between the two ROC curves. The resultant $p$ values of the two-tailed test are reported and values less than 0.01 are considered significant.
